# The Bloch point 3D topological charge induced by the magnetostatic interaction

**DOI:** 10.1038/s41598-021-01175-9

**Published:** 2021-11-05

**Authors:** F. Tejo, R. Hernández Heredero, O. Chubykalo-Fesenko, K. Y. Guslienko

**Affiliations:** 1 Instituto de Ciencia de Materiales de Madrid, Cantoblanco, 28049 Madrid, Spain; 2 Departamento de Matemática Aplicada a las TIC, ETSIST, Universidad Politécnica de Madrid, 28031 Madrid, Spain; 3 Departamento de Polímeros y Materiales Avanzados, Universidad del País Vasco, UPV/EHU, 20018 San Sebastián, Spain; 4 Ikerbasque, the Basque Foundation for Science, 48009 Bilbao, Spain

**Keywords:** Physics, Condensed-matter physics, Ferromagnetism

## Abstract

A hedgehog or Bloch point is a point-like 3D magnetization configuration in a ferromagnet. Regardless of widely spread treatment of a Bloch point as a topological defect, its 3D topological charge has never been calculated. Here, applying the concepts of the emergent magnetic field and Dirac string, we calculate the 3D topological charge (Hopf index) of a Bloch point and show that due to the magnetostatic energy contribution it has a finite, non-integer value. Thus, Bloch points form a new class of hopfions—3D topological magnetization configurations. The calculated Bloch point non-zero gyrovector leads to important dynamical consequences such as the appearance of topological Hall effect.

## Introduction

Recent advances of magnetic tomography, based on electron holography or X-ray magnetic circular dichroism, have allowed the first imaging of 3D magnetic configurations such as complex vortices, Bloch points and hopfions^1,2^. One of such 3D configurations is the Bloch point, a singularity in the magnetization distribution with a vanishing magnetization in the center. The occurrence of magnetization configurations consistent with the Bloch point configuration has been experimentally observed in several 3D nanostructures such as cylindrical magnetic nanowires^3^ and thick asymmetric circular dots^4^. Since the natural step forward in development of magnetic nanotechnology goes towards the third dimension^5^, the importance of these three-dimensional magnetization configurations will grow in time.

The Bloch point (hedgehog) is a kind of non-localized 3D magnetic soliton^6^. Similar to magnetic vortex, its energy diverges with increasing system size. This singularity^7^ can be also classified as a magnetic topological defect^8^. Solitons or nonlinear field configurations have been observed in many branches of physics such as classical field theory, fluid dynamics, optics, plasmas, liquid crystals, superconductivity, magnetism, etc. Solitons are solutions of nonlinear partial differential equations and they often have a topologically nontrivial nature, forming a subclass of topological solitons. Such topological solitons in higher spatial dimensions can be stable due to conservation of their topological charges, which are some integer values calculated as integrals from the field spatial distribution. The concepts and methods of algebraic topology are currently at the frontier of modern research in condensed matter physics. Sometimes nonlinear field configurations in ordered media bearing topological charges are called ¨topological defects¨ and homotopy theory provides the natural language for their classification.

Topological solitons of a vector field describing an order parameter can be classified by using maps from the coordinate space (${\varvec{r}}$) to the order parameter space. The vector order parameter for a ferromagnetic media is its net magnetization ${\varvec{M}}\left({\varvec{r}}\right)$. The magnetization field in 3D space represented by the unit field vector ${\varvec{m}}\left({r}_{\alpha }\right)={\varvec{M}}\left({\varvec{r}}\right)/\left|{\varvec{M}}\left({\varvec{r}}\right)\right|$ depends, in general, on three spatial coordinates ${r}_{\alpha }$, *α* = 1,2,3. The theory of topological charges in 1D and 2D space is well developed and used for the classification of topological magnetic solitons such as magnetic domain walls (kinks), vortices and skyrmions. Topological charges describe degrees of mappings (homotopy invariants) of 1D- (${S}_{1}$) or 2D coordinate space (${S}_{2}$) to the unit sphere ${{\varvec{m}}}^{2}=1$ in the magnetization space ${S}_{2}\left({\varvec{m}}\right)$, *i.e.*, ${{S}_{1}\to S}_{2}\left({\varvec{m}}\right),$ ${{S}_{2}\to S}_{2}\left({\varvec{m}}\right)$. A continuous vector field satisfying the condition ${{\varvec{m}}}^{2}=1$ is a degree of freedom of the nonlinear σ-model^9^ which describes properties of ferromagnets^10^ and antiferromagnets^6^ within the strong exchange interaction approximation. There is interest nowadays in 3D inhomogeneous magnetization configurations classified by a linking number of the preimages of two distinct points in ${S}_{2}\left({\varvec{m}}\right)$ on the 3D sphere ${S}_{3}$, *i.e*., by the Hopf index^7,11–13^. The corresponding configurations are called ¨hopfions¨. The Hopf index can be represented as some integral of the expression composed by a continuous field and its spatial derivatives^7^. The magnetization ${\varvec{m}}\left({\varvec{r}}\right)$ approaches some constant value ${{\varvec{m}}}_{0}$ at $\left|{\varvec{r}}\right|\to \infty$ for localized solitons, whereas ${\varvec{m}}\left({\varvec{r}}\right)$ is not uniform everywhere for non-localized solitons. The condition ${\varvec{m}}\left({\varvec{r}}\right)\to {{\varvec{m}}}_{0}$ implies that the Hopf index, which distinguishes the different homotopy classes ${\pi }_{3}\left({S}_{2}\right)=Z$, is an integer in infinite samples^9^. Therefore, there is a class of hopfions (toroidal hopfions or twisted loops of the skyrmion strings) in ordered media, which are described by an integer Hopf index ${Q}_{H}=0, \pm 1, \dots$^9,14–21^. The Hopf index of the toroidal hopfions is a product of two winding numbers, the planar winding and the twisting of the magnetization configuration, respectively. The simplest magnetic toroidal hopfions with $\left|{Q}_{H}\right|=1$ were considered in infinite films^17,18^ and cylindrical dots^15,16,19,20^ using the unit-vector field ansatz^22^. Existence of the stable static hopfions was recently demonstrated experimentally in soft condensed matter media—liquid crystals^21^ and chiral colloidal ferromagnets^14^ through direct 3D imaging and simulations. It was shown numerically that the toroidal hopfions with $\left|{Q}_{H}\right|=1$ can be the ground state of circular chiral nanodots^20^ assuming a strong surface magnetic anisotropy, if the magnetostatic energy and bulk magnetic anisotropy are neglected.

Previously, the concept of Bloch point suggested in refs.^23,24^ was extensively used to describe static and dynamic properties of the bubble domains^25^. Later, the Bloch points were experimentally detected in the garnet films^26,27^. The concept of Bloch point was used to explain the vortex core (Bloch line) reversal induced by the static^28^ out-of-plane or oscillating in-plane magnetic field^29^ in the vortex state dots and 3D ¨bobber¨ configurations in magnetic films with Dzyaloshinskii-Moriya interaction^30^. However, the hypothesis^31^ that standing waves of the vortex core line oscillation have Bloch points at the nodes was not confirmed by the detailed calculations in ref.^32^ of the vortex gyromode profiles. In many recent articles^1–4,26–29,33–36^ the Bloch points are also considered as topological defects of the 3D magnetization vector field. Typically, those defects are characterized by its non-zero gyrovector flux over some closed surface in the 3D space^1,4,33^. This property is very useful since the gyrovector flux represents the net number of topological defects (Bloch points) enclosed within a given volume. However, we note that unlike the 2D case, this value does not coincide with a 3D topological charge (Hopf index) given by the 3D homotopy theory^7^. Particularly, it does not allow to distinguish between topologically non-trivial and trivial magnetic singularities. In this article we consider in detail a topological charge of a 3D magnetization configuration known as hedgehog or Bloch point, which allows their classification as topologically trivial or non-trivial magnetization configuration.

We adopt the point of view that any magnetization configuration with a non-zero Hopf index is actually topologically non-trivial (i.e., homotopically distinct from the saturated magnetization state) and is, therefore, a magnetic hopfion. We show that, except the toroidal hopfions, there is another class of magnetization configuration (non-localized solitons), for which the value of the Hopf index ${Q}_{H}$ is finite, but not an integer. An example of this is a spiral Bloch point configuration, which now can be classified as a magnetic hopfion different from the usual toroidal one. Note that while 2D skyrmion/3D toroidal hopfion have topological charge one, a pure 2D magnetic vortex state has topological charge 1/2 and other fractional topological charges also exist in the quantum field theories^37^. In the quantum field theories, they appear for the ground states characterized by kink-type solitons with non-zero boundary conditions at infinity and they are responsible for the quantum fractional Hall conductivity. Also, the fractional topological charge is a widely used concept for, e.g., optical vortices^38^ or liquid crystals where they are induced by the presence of boundaries^8^. Here, we show that while for a simple hedgehog magnetization configuration (radial Bloch point) the Hopf index is zero, the influence of magnetostatic energy for a spiral Bloch point inside a sphere leads to a small non-zero 3D topological charge, making this configuration topologically non-trivial. This allows to distinguish different 3D magnetic configurations by their topological charge.

## Results

We consider a spherical ferromagnetic particle with the radius $R$. We use a spherical coordinate system with polar Θ and azimuthal Φ angles to describe the unit magnetization vector ${\varvec{m}}\left({\varvec{r}}\right)={\varvec{m}}\left(\Theta \left({\varvec{r}}\right),\Phi \left({\varvec{r}}\right)\right)$ and the radius vector ${\varvec{r}}=\left(r,\vartheta ,\varphi \right)$.

A general expression of the Hopf invariant for the mapping of the spheres $f:{S}_{3}\to {S}_{2}$ can be found in ref.^9^

1 $${Q}_{H}=-\frac{1}{{\left(4\pi \right)}^{2}}\int dV{\varvec{A}}\bullet {\varvec{B}},$$

where the field ***B ***coincides with the gyrovector density^39,40^ related to the inhomogeneous magnetization ${\varvec{m}}\left({\varvec{r}}\right)$

2 $${\varvec{B}}=\mathrm{sin}\left(\Theta \right)\nabla\Theta \times \nabla\Phi .$$

The field $\mathbf{B}$ is called the emergent magnetic field^16–19,39–41^ although it is not the real induction field. The corresponding vector potential ${\varvec{A}}$ of the emergent magnetic field is defined as ${\varvec{B}}=\nabla \times {\varvec{A}}$ and is, therefore, subject to a gauge choice. The Hopf index is then calculated as the integral over the system volume^7,11^. It has been rigorously proved that this integral is the homotopy invariant of the mapping *f* for twice differentiable manifolds. To secure gauge invariance of the integral (1), the emergent magnetic field $\mathbf{B}\left(\mathbf{r}\right)$ has to satisfy the condition $\nabla \bullet \mathbf{B}=0$ (no magnetic monopoles) and to vanish sufficiently fast at $\left|{\varvec{r}}\right|\to \infty$. Therefore, strictly speaking, the Hopf index defined by Eq. (1) is gauge invariant only for infinite samples.

Feldkeller^23^ suggested the following magnetization ansatz to describe the Bloch point: $\Theta \left({\varvec{r}}\right)=\vartheta$ or $\Theta \left({\varvec{r}}\right)=\pi -\vartheta$, and $\Phi \left({\varvec{r}}\right)=\pm \varphi +\gamma$. This ansatz, introducing explicitly the Bloch point polarization *p* =  ± *1* and integer winding number *q*, can be written in the form^33^.

3 $$\Theta \left({\varvec{r}}\right)=p\vartheta +\frac{\pi }{2}\left(1-p\right),\Phi \left({\varvec{r}}\right)=q\varphi +\gamma .$$

The ansatz (3) is similar to the ansatz used for toroidal hopfions^11,12^, where $\gamma$ has the sense of the twisting angle around the torus ring. The magnetization components corresponding to the ansatz (3) at *q* = 1 are

4 $${m}_{r}={sin}^{2}\vartheta \mathrm{cos}\gamma +p{cos}^{2}\vartheta , {m}_{\vartheta }=sin\vartheta \mathrm{cos}\vartheta \left(\mathrm{cos}\gamma -p\right), {m}_{\varphi }=\mathrm{sin}\vartheta \mathrm{sin}\gamma.$$

**Figure 1:**
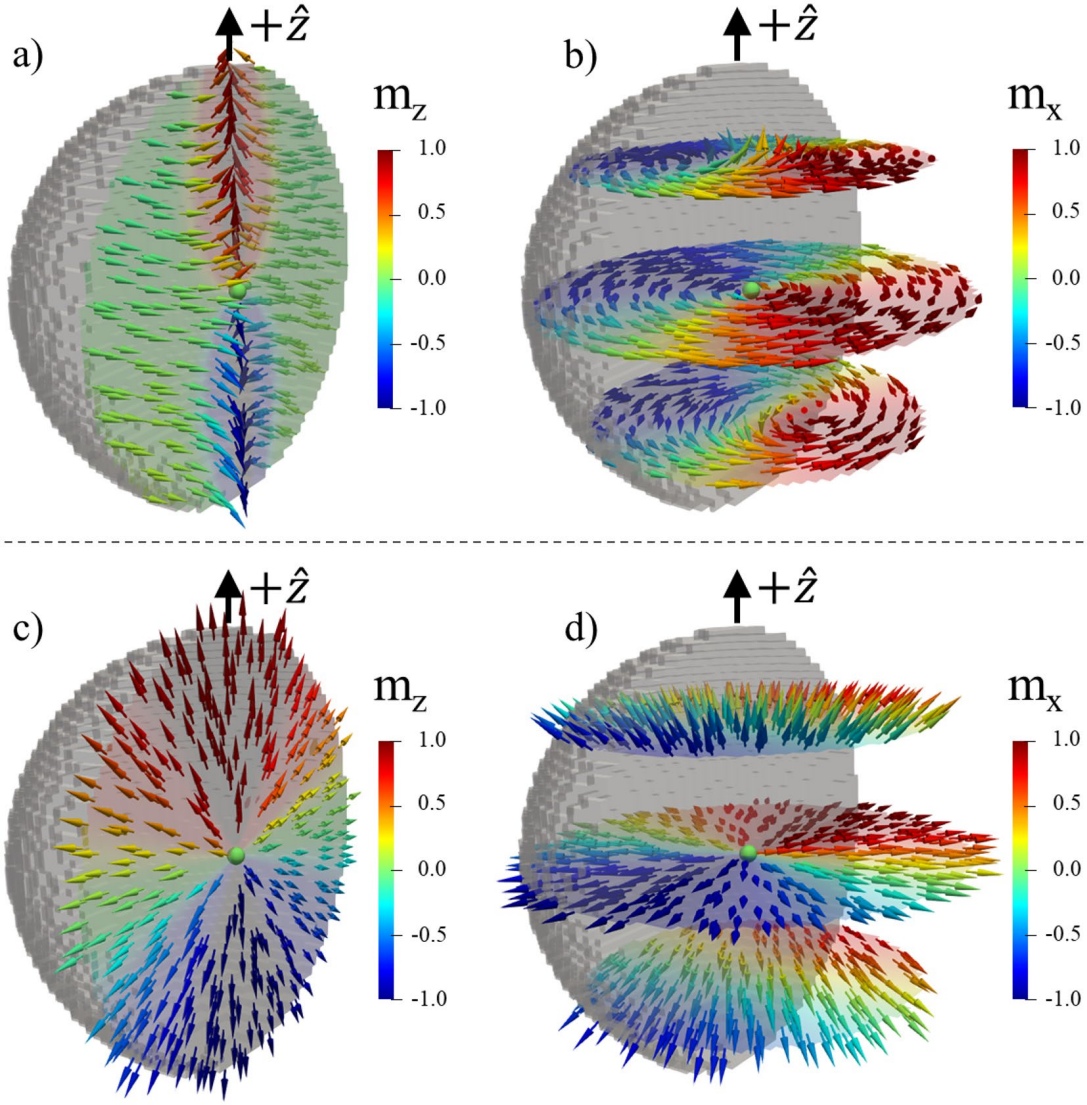
Simulated images of the Bloch points. **(a,b)** The spiral Bloch point **(b)** with the simulated inhomogeneous helicity $\gamma \left({\varvec{r}}\right)$ and polarization *p* =  + 1 in a spherical nanoparticle with radius *R* = 50 nm containing a Bloch point in the origin. (**c–d**) The radial hedgehog Bloch point with the helicity $\gamma \left({\varvec{r}}\right)=0$ and polarization *p* =  + 1. The magnetization configurations were obtained by micromagnetic simulations with parameters mentioned in the main text. The hedgehog Bloch point **(c,d)** was stabilized with no magnetostatic interactions. Magnetic configurations in the vertical **(a,c)** and transverse **(b,d)** cross sections are presented by arrows colored by the magnetization component indicated by the color map.

The azimuthal angle (helicity) *γ* can be a function of the radius vector **r**. We assume that the helicity $\gamma =\gamma \left(r,\vartheta \right)$ to keep the axial symmetry with respect to the spherical coordinate axis *Oz*. Different values of the integers *p*, *q* and functions γ describe different kinds of the Bloch points. For instance, the set *q* = 1, $\mathrm{cos}\gamma =p$ describes the simplest radial hedgehog Bloch point with the magnetization ${\varvec{m}}=p\widehat{{\varvec{r}}}$. Non-trivial helicity angles $\gamma$ ($\gamma \ne 0,\pi$) describe ¨spiral¨ or ¨vortex¨ Bloch points. The calculated magnetization configurations of the different Bloch points are shown in Fig. 1.

We calculate the emergent magnetic field and vector potential for the particular case of the Bloch point described by the ansatz (3) as

5 $${{\varvec{B}}}_{0}=pq\frac{\widehat{{\varvec{r}}}}{{r}^{2}}-p\frac{\partial \gamma }{\partial r}\mathrm{sin}\left(\vartheta \right)\frac{\widehat{\boldsymbol{\varphi }}}{r},\boldsymbol{ }{\varvec{A}}={A}_{r}\widehat{{\varvec{r}}}-\frac{pq}{r}\frac{1+\mathrm{cos}\left(\vartheta \right)}{\mathrm{sin}\left(\vartheta \right)}\widehat{\boldsymbol{\varphi }},$$

where $\widehat{{\varvec{r}}}$ and $\widehat{\boldsymbol{\varphi }}$ are the unit vectors along the radial and azimuthal directions, respectively.

Note that the Bloch point can be treated as a Dirac magnetic monopole—source of the emergent magnetic field with a charge *pq* localized at the origin of the coordinate system *r* = 0^1,4,33^. The resulting emergent field (Eq. 5) is also divergent at r = 0 and thus does not satisfy the condition $\nabla \bullet {\varvec{B}}=0$, which is necessary for the Hopf index gauge invariance. To circumvent this problem, we use the Dirac’s approach to the singularities in the electromagnetic field^42^, introducing an effective “anti-monopole” at the infinity with a charge opposite to the charge of the monopole $\nabla \bullet {{\varvec{B}}}_{0}$. A correct distributional derivation introduces an extra singular term (a Dirac string) in the emergent field ***B***, ${{\varvec{B}}}_{s}\left({\varvec{r}}\right)=-4\pi pqH\left(z\right)\delta \left(x\right)\delta \left(y\right)\widehat{{\varvec{z}}}$, which secures the condition $\nabla \bullet {\varvec{B}}=0$ for the sum of both fields and allows to remove the singularity at *r* = 0. Here $H\left(z\right)$ is the Heaviside step function. The total emergent magnetic field in Eq. (1) is then ${\varvec{B}}={{\varvec{B}}}_{0}+{{\varvec{B}}}_{s}$. The gauge was chosen with the vector potential ***A*** singular along the positive *z*-axis of the spherical coordinate system (the chosen Dirac string is located along the semi-axis *z* > 0). The ${A}_{r}$ component is determined by the equation $\frac{\partial {A}_{r}}{\partial \vartheta }=p\frac{\partial \gamma }{\partial r}\mathrm{sin}\left(\vartheta \right)$. It is physically reasonable to use the assumption ${A}_{\vartheta }=0$ for the Dirac-like vector potential (Eq. 5), especially accounting that the ${A}_{\vartheta }$ component does not contribute to the Bloch point Hopf index (Eq. 2). Since the ¨fictious¨ emergent magnetic field and vector potential satisfy now the Maxwell equation $\nabla \bullet {\varvec{B}}=0$ and the relation ${\varvec{B}}=\nabla \times {\varvec{A}}$, they obey the same classical electrodynamics as real magnetic fields.

The global gyrovector defined as ${\varvec{G}}=\int dV{\varvec{B}}$ differs from zero due to the Dirac string contribution to the emergent field ***B***. The gyrovector of the Bloch point embedded in a sphere of radius *R* is ${\varvec{G}}=-4\pi pqR\widehat{{\varvec{z}}}$ and it does not depend on the helicity $\gamma$. However, importantly it depends on the sphere radius *R.* This non-zero gyrovector of the Bloch point results, in particular, in a gyroforce perpendicular to the Bloch point velocity (topological Hall effect) similar to that of 2D magnetic topological solitons, vortices and skyrmions. The flux of the emergent field $\oint d{\varvec{S}}\bullet {\varvec{B}}$ through the surface surrounding the origin (the BP center) is not a topological charge as it was in the case of 2D magnetization textures ${\varvec{m}}\left(x,y\right)$, where $\left(1/4\pi \right)\int d{\varvec{S}}\bullet {\varvec{B}}=pq$ is an integer number (skyrmion number), and *S* is a surface $z=const$. Moreover, the total flux is $\oint d{\varvec{S}}\bullet {\varvec{B}}=0$, but note that $\oint d{\varvec{S}}\bullet {{\varvec{B}}}_{0}=-\oint d{\varvec{S}}\bullet {{\varvec{B}}}_{s}=4\pi pq \ne 0$, so that the well-known^25^ and widely-used result is reproduced. We underline that the Hopf index density of a 3D magnetization configuration ${\varvec{m}}\left({\varvec{r}}\right)$ is a non-local function of the gyrovector density ***B*** and, therefore, it cannot be written in such simple local form as 2D topological charge density.

It is evident from Eq. (5) that the spatial dependence of the angle $\gamma =\gamma \left({\varvec{r}}\right)$ is of principal importance for the calculation of the Hopf index of a Bloch point. Substituting Eq. (5) into Eq. (2) and conducting the integration over a sphere with the radius *R,* we get the expression for the Hopf index

6 $${Q}_{H}=-\frac{q}{4\pi }{\int }_{0}^{\pi }d\vartheta \mathrm{sin}\vartheta \left(1+\mathrm{cos}\vartheta \right)\left[\gamma \left(R,\vartheta \right)-\gamma \left(0,\vartheta \right)\right],$$

which is the main result of the present article. It is evident that a simple radial hedgehog solution with $\gamma =0,\pi$ has zero Hopf index and in this sense is topologically trivial (despite of the non-zero value of the gyrovector flux $(\oint d{\varvec{S}}\bullet {\varvec{B}}$). In other words, the gyrovector flux itself is not sufficient to characterize the topological properties of the Bloch points and introducing the 3D topological charge (1) is justified.

The Bloch point soliton is not localized in space (the magnetization is not homogenous far from the origin). Therefore, the value of ${Q}_{H}$ given by Eq. (6) can be a non-integer number, whereas for the particular case of the localized toroidal 3D solitons ${Q}_{H}$ is always an integer. The equilibrium function $\gamma \left({\varvec{r}}\right)$ can be determined from the total magnetic energy variation $\delta W/\delta \gamma =0.$ The energy density within the simplest realistic model of a soft magnetic nanoparticle consists of the exchange and magnetostatic contributions

7 $$w=A\left[{\left(\nabla\Theta \right)}^{2}+{\mathrm{sin}}^{2}\Theta {\left(\nabla\Phi \right)}^{2}\right]-\frac{1}{2}{M}_{s}{\varvec{m}}\bullet {\varvec{H}},$$

where *A* is the exchange stiffness, ${M}_{s}$ is the saturation magnetization and ${\varvec{H}}$ is the magnetostatic field which is a functional of the magnetization ${\varvec{m}}\left({\varvec{r}}\right)$.

The exchange energy density ${w}_{ex}=A\left[\left(1+{q}^{2}\right)/{r}^{2}+{sin}^{2}\vartheta {\left(\nabla \gamma \right)}^{2}\right]$ is an increasing function of the Bloch point vorticity $\left|q\right|$. The lowest energy magnetization configurations correspond to $q=0,\pm 1$. The configuration with *q* = 0 is not a Bloch point and has Hopf index ${Q}_{H}=0$. ¨Anti-Bloch point¨ configurations with $q=-1$ are not considered here. Therefore, we consider in more detail the case *q* = 1, which corresponds to an axially symmetric Bloch point described by the ansatz (3). The variation of the total magnetic energy $W=\int dVw$ leads to the integro-differential equation for the function $\gamma \left({\varvec{r}}\right)$

8 $${l}_{e}^{2}\left[{\nabla }^{2}\gamma +\frac{2\mathrm{cot}\vartheta }{{r}^{2}}\frac{\partial \gamma }{\partial \vartheta }\right]=\mathrm{sin}\gamma \left({h}_{r}+{h}_{\vartheta }\mathrm{cot}\vartheta \right),$$

where ${l}_{e}=\sqrt{A/2\pi {M}_{s}^{2}}$ is the exchange length serving as a natural spatial scale, and ${\varvec{h}}={\varvec{H}}/{4\pi M}_{s}$ is the magnetostatic field, see “Methods” 

**Figure 2:**
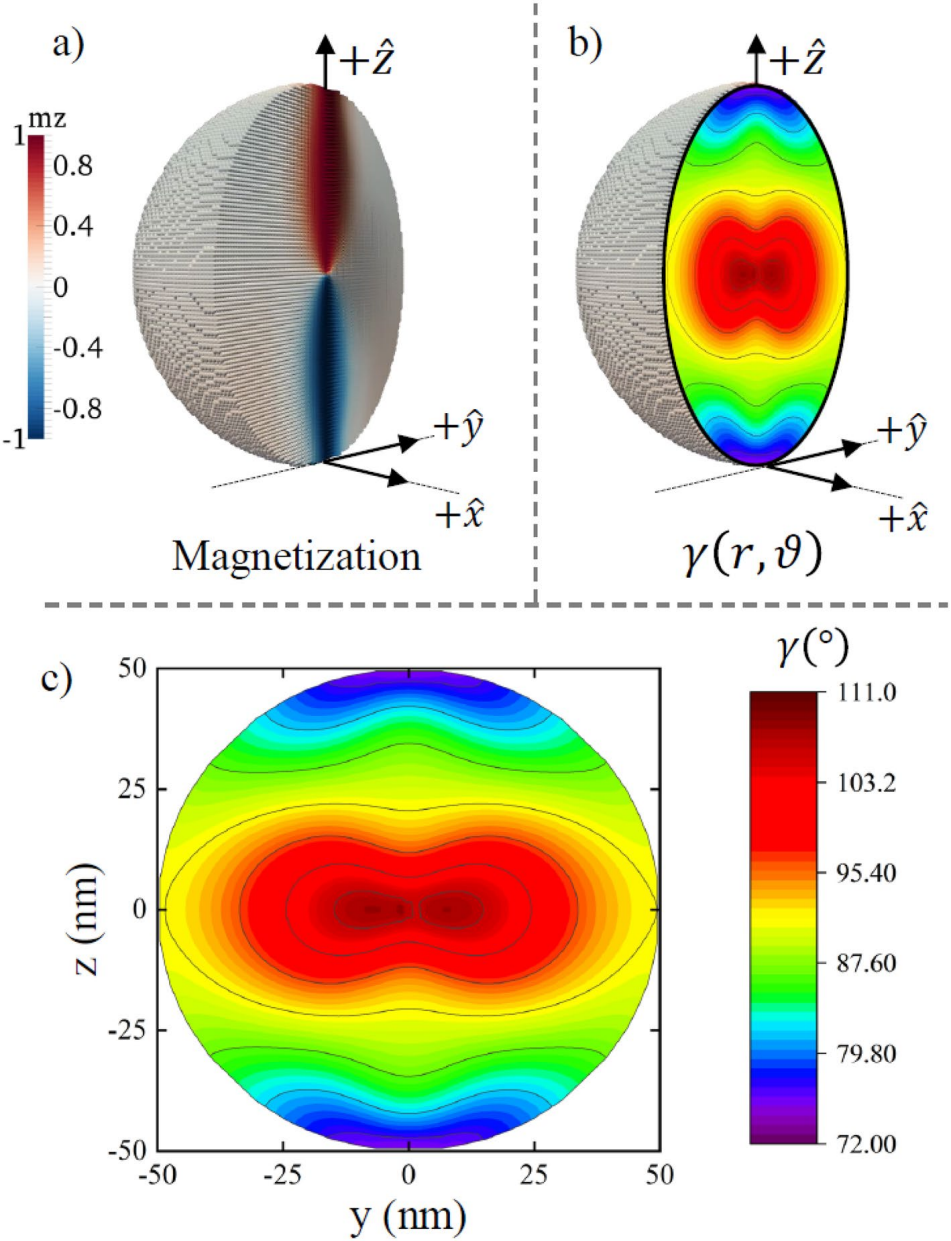
Bloch point magnetization configuration. The magnetization configuration **(a)** (${m}_{z}$ component) and helicity $\gamma \left({\varvec{r}}\right)$ **(b,c)** of a spherical nanoparticle with radius *R* = 100 nm containing a Bloch point in the origin.

**Figure 3:**
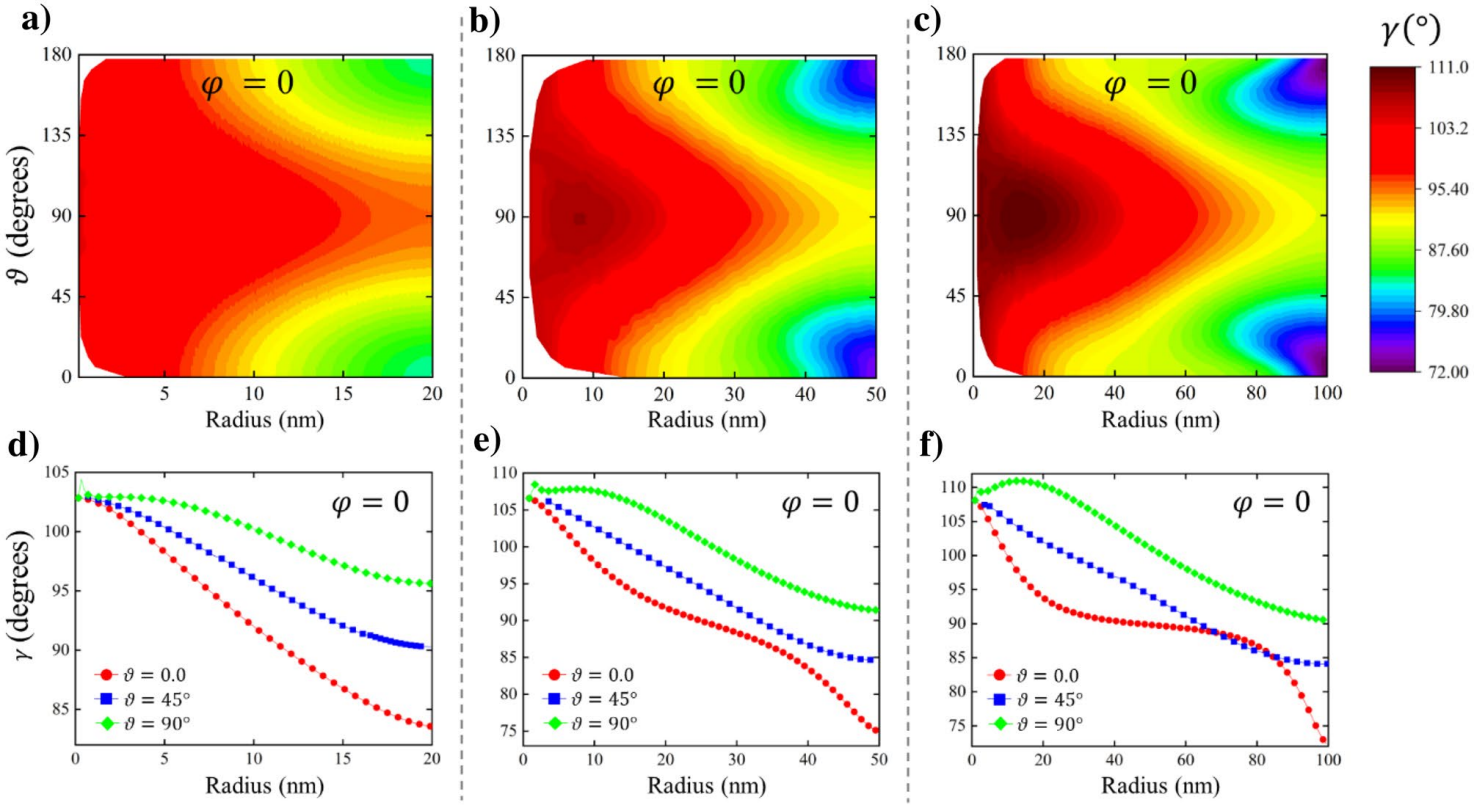
Helicity of a Bloch point. The helicity $\gamma \left(r,\vartheta \right)$ maps of the spherical magnetic nanoparticles of different radii containing a Bloch point: **(a)** *R* = 20 nm, **(b)** *R* = 50 nm, **(c)** *R* = 100 nm. The radial dependences of the helicity $\gamma \left(r,\vartheta \right)$ at the fixed spherical angle $\vartheta$ for *R* = 20 nm **(d)**, 50 nm **(e)** and 100 nm **(f)**.

**Figure 4:**
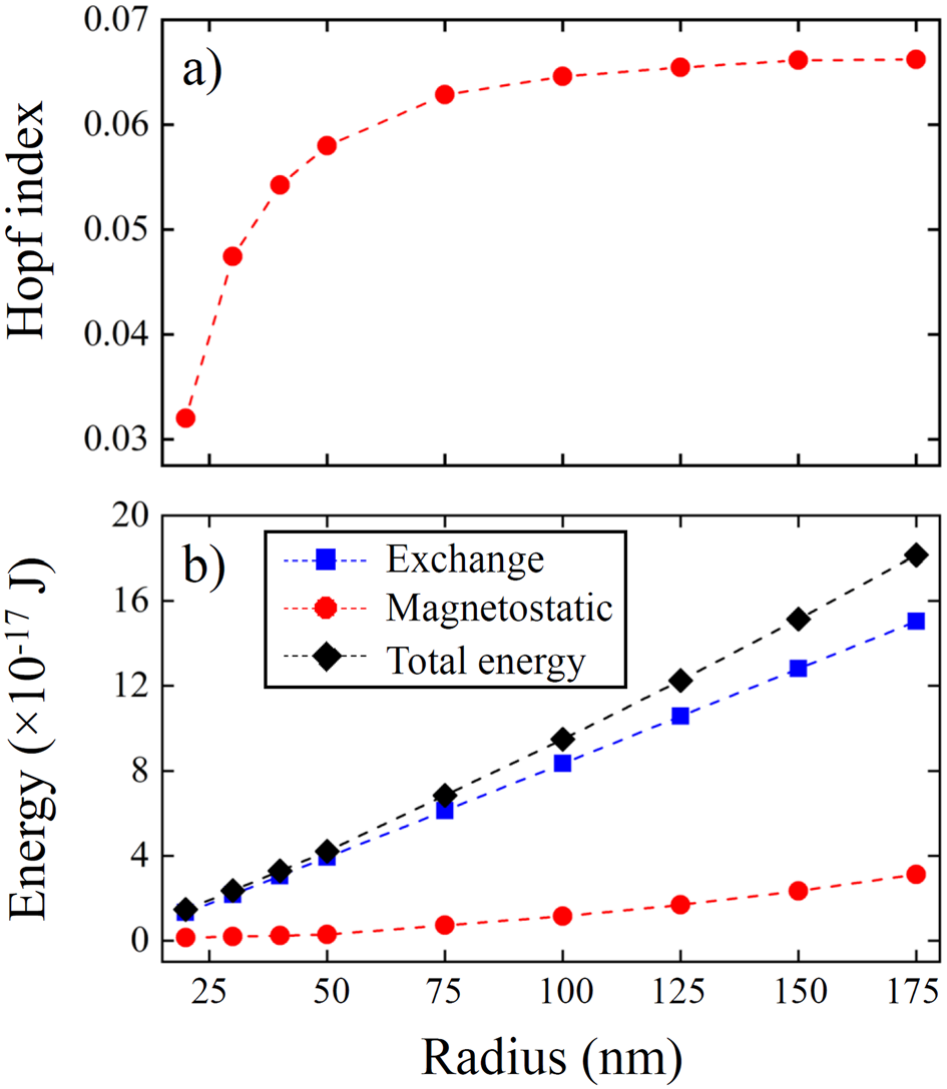
Hopf index and magnetic energy of a Bloch point. The calculated Hopf index **(a)** and the energy **(b)** of a Bloch point in a spherical magnetic nanoparticle as function of the particle radius *R*.

To calculate numerically the Hopf index of a Bloch point in a soft magnetic sphere of radius $R$, we conducted micromagnetic simulations. We used the material exchange stiffness $A=$ 21 pJ/m and the saturation magnetization ${M}_{s}=$ 1700 kA/m with a small discretization size (≤ 1 nm, see “Methods” for details). The simulations allow to evaluate the helicity angle $\gamma \left(r,\vartheta \right)$ (Figs. 2 and 3) confirming that it is essentially non-uniform and that for a particular Bloch point with *p* =  + 1 it decreases with the distance from the center. The Hopf index was numerically evaluated from Eq. (6) leading to a non-zero value (Fig. 4a), which increases with the nanosphere radius increasing. Note that the Bloch point with *p* = − 1 has different values of $\gamma \left(r,\vartheta \right)$, increasing with the distance from the center but with maintaining the difference $\left|\gamma \left(R,\vartheta \right)-\gamma \left(0,\vartheta \right)\right|$, and thus the Hopf index is the same in magnitude but opposite in sign.

## Discussion

The solutions of Eq. (8) depend only on one dimensionless parameter $R/{l}_{e}$. We note that the energy density (Eq. 7) and corresponding equilibrium Eq. (8) are essentially more complicated than those in the Faddeev–Skyrme model^43^, where terms quartic in spatial derivatives of the field ${\varvec{m}}\left({\varvec{r}}\right)$ are included. If only the exchange interaction is included in the energy density *w*, then $\partial \gamma /\partial {\varvec{r}}=0$, $\gamma \left({\varvec{r}}\right)=const$ and the Hopf index of any Bloch point is equal to zero. If the magnetostatic energy is accounted, then there is only one trivial homogenous solution of the equilibrium Landau-Lifshitz Eq. (8), which corresponds to the hedgehog Bloch point ($\mathrm{cos}\left(\gamma \right)=p, \gamma =0,\pi$) with ${Q}_{H}=0$. This hedgehog solution corresponds to the maximum of the total energy^33,35^. All other solutions $\gamma =\gamma \left({\varvec{r}}\right)$ are inhomogeneous and yield finite values of the Hopf index ${Q}_{H}$. The Eq. (8) for $\gamma \left({\varvec{r}}\right)$ is a non-linear integro-differential equation and cannot be solved analytically, except some simple limiting cases. The ansatz $\gamma \left({\varvec{r}}\right)=\gamma =\mathrm{const}$ can be considered as a rough trial function. The corresponding magnetostatic energy calculated using Eq. (4) is the simple function of *γ*, ${W}_{m}\left(\gamma \right)=\left(4\pi {M}_{s}^{2}V\right)\left(2/15\right)\left[3/4+p\mathrm{cos}\gamma +2{\mathrm{cos}}^{2}\gamma \right]$ having the minima at $\mathrm{cos}{\gamma }_{0}=-p/4$, *i.e.,* ${\gamma }_{0}=104.5^\circ$ at *p* =  + 1 and ${\gamma }_{0}=75.5^\circ$ at *p* = − 1^33^. The exchange energy of the BP non-localized solitons, ${W}_{ex}\sim R$, and the magnetostatic energy, ${W}_{m}\sim {R}^{3}$, diverge at $R\to \infty$. Therefore, the sphere radius should be finite. On the other hand, the sphere radius has to be large enough, $R\gg {l}_{e}$, to secure the gauge invariance of the Hopf index (Eq. 2).

It was assumed in previous calculations of the Bloch point magnetostatics that the helicity $\gamma \left({\varvec{r}}\right)=\mathrm{const}$. The authors of ref.^33^ accounted only for the dependence $\gamma \left(r\right)$. However, $\gamma \left({\varvec{r}}\right)$ cannot be function only on the radial coordinate *r*, as one can immediately see from Eq. (8). It has to be at least a function of *r* and the polar angle ϑ. Even if we assume that $\gamma =\gamma \left(r\right)$, the magnetostatic energy ${W}_{m}=-\left({M}_{s}^{2}/2\right)\int dV{\varvec{m}}\bullet {\varvec{h}}$ is a double-volume integral of the magnetization components and thus the variational equation is integro-differential, like Eq. (8). The dependence of the angle $\gamma \left(r,\vartheta \right)$ on both arguments *r* and *ϑ* was confirmed by atomistic simulations^33^. The simulated angle $\gamma \left(r,\vartheta \right)$ is relatively small and its dependence on *ϑ* is weak. That can be explained by the small sphere radius used $R=18a$ (*a* is the cubic lattice period). The value of $R$ is $\approx 5-6$ nm for the typical values of *a*, *i.e.,* it is comparable with the exchange length ${l}_{e}$ (typically ${l}_{e}\approx$ 5 nm) and the exchange energy dominates.

The variable helicity angle $\gamma \left({\varvec{r}}\right)$ is within the range $\left[\mathrm{0,2}\pi \right]$, therefore the difference $\gamma \left(R,\vartheta \right)-\gamma \left(0,\vartheta \right)$ does not exceed $2\pi$. Substituting $2\pi$ into Eq. (6) we get $\left|{Q}_{H}\right|=1$, a value that can be considered as an upper bound for the Hopf index of the Bloch points with the winding number $q=1$. For a solution $\gamma \left({\varvec{r}}\right)$ of Eq. (8) the value of the Hopf index is finite and satisfies inequality $\left|{Q}_{H}\right|\le 1$. The exchange energy dominates at $r\to 0$ ($r\ll {l}_{e}$), therefore the angle $\gamma \left({\varvec{r}}\right)\approx \mathrm{const}$ at $r\to 0$. Accounting for a small magnetostatic energy removes the degeneracy with respect to the angle $\gamma \left({\varvec{r}}\right)$ and results in an equilibrium value $\gamma \left({\varvec{r}}\right)={\gamma }_{0}={\mathrm{cos}}^{-1}\left(-p/4\right)$ at $r\to 0$. If we make the additional assumption that in the limit $R\to \infty$ $\gamma \left(R,\vartheta \right)\to 0$ at $p=+1$ or $\gamma \left(R,\vartheta \right)\to \pi$ at $p=-1$, then the roughly estimated Hopf index is ${Q}_{H}=-\left[{\mathrm{cos}}^{-1}\left(p\right)-{\mathrm{cos}}^{-1}\left(-p/4\right)\right]/2\pi =p {\mathrm{cos}}^{-1}\left(-1/4\right)/2\pi$, or ${Q}_{H}=0.290p$. Using the numerical data on the dependence $\gamma \left(r,\vartheta \right)$ in ref.^33^, Eq. (6) yields the estimated value of ${Q}_{H}\approx 0.080$ for $p=+1$ for a small simulated sphere of radius $R\approx 5-6$ nm. As we showed above (Fig. 4a) the Hopf index ${Q}_{H}$ of the Bloch point in a spherical ferromagnetic particle is smaller than the value extracted from simulations^33^ and the function ${Q}_{H}\left(R\right)$ increases with *R* increasing because the influence of the magnetostatic interaction, which is responsible for the finite values of ${Q}_{H}$, increases with radius *R* increasing.

The simulations also confirmed that the exchange energy is ${W}_{ex}\sim R$ (Fig. 4b). The simulated slope is ${W}_{ex}/R$ =8.57 10^–19^ J/nm, whereas the slope ${W}_{ex}/R$ calculated assuming uniform helicity $\gamma \left({\varvec{r}}\right)=const$ is 5.28 10^–19^ J/nm. The simulated value of ${W}_{m}$ is 3.0 10^–17^ J for *R* = 175 nm, whereas that calculations assuming $\gamma \left({\varvec{r}}\right)=\mathrm{const}$ give 6.79 10^–17^ J. The simulated increase of the exchange energy and reduction of the magnetostatic energy of the spiral Bloch point in comparison with the uniform helicity $\gamma \left({\varvec{r}}\right)$ case along with the direct helicity simulations (Figs. 2 and 3) confirm that the helicity angle $\gamma \left({\varvec{r}}\right)$ is essentially non-uniform.

Recently hedgehog-like 3D magnetization configurations with a non-integer Hopf index were observed in dome-shaped soft magnetic (permalloy) dots using magnetic force microscopy^44^. Nowadays, development of soft X-ray tomography allows to observe 3D magnetization configurations of thin films and dots (including the Bloch points) with a resolution of one nanometer^1,2^. Very recently specific 3D topological magnetization configurations, vortex rings, were detected using this experimental technique^45^. The Bloch points were identified^45^ by an abrupt change of the emergent magnetic field ${{\varvec{B}}}_{0}$ direction and the Bloch points ($q=+1$) and anti-Bloch points ($q=-1$) were distinguished by positive and negative signs of the divergence $\nabla \bullet {{\varvec{B}}}_{0}$. Therefore, our calculations of the magnetization configurations of Bloch points in spherical dots, their Hopf index and gyrovector, can be applied to interpret future experiments in imaging topologically non-trivial 3D magnetization configurations.

In summary, we applied the concept of Dirac monopole for the emergent magnetic field to calculate the Bloch point 3D topological charge (Hopf index). Using an inhomogeneous helicity of the Bloch point magnetization we showed analytically and confirmed by simulations that the Hopf index has some finite, non-integer value determined by the magnetization configuration of the Bloch point, non-localized topological soliton. Bloch points form a new class of 3D magnetization configurations, whereas traditional toroidal hopfions (localized topological solitons) considered before have an integer Hopf index. One of the important consequences of this approach, that can be tested experimentally, is a non-zero gyrovector of the Bloch point resulting in its non-trivial dynamics, when the direction of motion is not parallel to a driving force (topological Hall effect). The volume averaged emergent magnetic field (the gyrovector) was calculated in the recent paper by Kanazawa et al.^46^ for skyrmion lattice magnetization distribution in MnGe containing the monopole/anti-monopole pairs (Bloch/anti-Bloch point pairs) and was used to interpret the observed topological Hall resistivity.

Our last remark refers to the fact that the non-zero non-integer topological charge is induced in our case by the sample boundary and particularly by the minimization of the magnetostatic energy at the surface of the sphere. Therefore, this is an intrinsically finite-size effect. A similar effect has been observed in liquid crystals for the boojums structures^8^ where the particle geometry causes distortions of the vector field and consequently mapping onto a 2D sphere does not fully cover it. At the same time, we should stress that the notion of the topological invariance from a pure mathematical point of view is not applicable for finite-size systems since the defect can always escape through the system border. However, this notion allows the classification of different topological defects according to their topological charge values. Particularly, we can distinguish between the radial hedgehog (trivial structure) and the spiral Bloch point (topologically non-trivial structure) while the gyrovector flux approach gives the same value. The role of 3D topological charge (Hopf index) in the 3D soliton equations of motion of toroidal and Bloch point hopfions is still unclear. We believe that such role will be clarified in the nearest future. The first steps in this direction were done in the recent papers^17–19^.

## Methods

### Details of the calculations of the magnetostatics

The explicit form of the magnetostatic field components used in Eq. (8) of the main text is

M1 $${h}_{\alpha }\left(r,\vartheta \right)=\int dV^{\prime}{g}_{\alpha \beta }\left(r,\vartheta ,r^{\prime},\vartheta ^{\prime}\right){m}_{\beta }\left(r^{\prime},\vartheta ^{\prime}\right),{g}_{\alpha \beta }\left(r,\vartheta ,r^{\prime},\vartheta ^{\prime}\right)=\frac{1}{2\pi }\int d\varphi \int d\varphi ^{\prime}{G}_{\alpha \beta }\left({\varvec{r}},{\varvec{r}}^{\prime}\right),$$

where the magnetostatic kernels are ${G}_{\alpha \beta }\left({\varvec{r}},{\varvec{r}}^{\prime}\right)=-{\left(4\pi \right)}^{-1}{\left({\nabla }_{\varvec{r}}\right)}_{\alpha }{\left({\nabla }_{{\varvec{r}}^{\prime}}\right)}_{\beta}{\left|{\varvec{r}}-{\varvec{r}}^{\prime}\right|}^{-1}$, $\alpha ,\beta =r,\vartheta$.

The magnetostatic field components can be obtained from the scalar magnetostatic potential $\psi$, ${\varvec{h}}=-{\left(4\pi \right)}^{-1}\nabla \psi$, where the potential *ψ* of a Bloch point is calculated as

M2 $$\psi \left(r,\vartheta \right)=\pi pr\left(1+{\mathrm{cos}}^{2}\vartheta \right)+\int dr^{\prime}{r^{\prime}}^{2}\int {\mathit{d}}{\vartheta }^{\prime}\mathrm{sin}\vartheta^{\prime}\Gamma \left(r,\vartheta ,r^{\prime},\vartheta^{\prime}\right)\mathrm{cos}\gamma \left(r^{\prime},\vartheta^{\prime}\right),$$

$$\Gamma \left(r,\vartheta ,r^{\prime},\vartheta ^{\prime}\right)={\mathrm{sin}}^{2}\vartheta ^{\prime}\left(\frac{\partial }{\partial r^{\prime}}+\frac{\mathrm{cot}\vartheta ^{\prime}}{r^{\prime}}\frac{\partial }{\partial \vartheta ^{\prime}}\right)F\left(r,\vartheta ,r^{\prime},\vartheta ^{\prime}\right),$$

where the function $F\left(r,\vartheta ,r^{\prime},\vartheta ^{\prime}\right)=2\pi {\sum }_{l}{f}_{l}\left(r,r^{\prime}\right){P}_{l}\left(\mathrm{cos}\vartheta \right){P}_{l}\left(\mathrm{cos}\vartheta ^{\prime}\right)$, ${P}_{l}\left(x\right)$ are the Legendre polynomials, ${f}_{l}\left(r,r^{\prime}\right)={r}_{<}^{l}/{r}_{>}^{l+1}$, ${r}_{<}=\mathrm{min}\left( {r}_{<}, {r}_{>}\right),$ ${r}_{>}=\mathrm{max}\left( {r}_{<}, {r}_{>}\right)$, and $l=\mathrm{0,1},2,\dots$^47^.

### Micromagnetic simulations

The assumption of the continuous micromagnetic approach that the magnetization can be described by a continuous vector field ${\varvec{M}}\left({\varvec{r}}\right)$ with a constant absolute value $\left|{\varvec{M}}\left({\varvec{r}}\right)\right|={M}_{s}$ |(${M}_{s}$ is the saturation magnetization) is not valid in the vicinity of some singular points (Bloch points), where the magnetization is undefined^23,24,28^. The magnetization ${\varvec{M}}\left({\varvec{r}}\right)$ can be completely described by the unit vector field ${\varvec{m}}\left({\varvec{r}}\right)$ only if ${M}_{s}\left({\varvec{r}}\right)=const.$ The assumption that ${M}_{s}\left({\varvec{r}}\right)$ is constant is violated at distances of $\approx$ 1 nm from a point singularity^35^, which in the exchange approximation is reduced to the condition ${M}_{s}\left({\varvec{r}}=0\right)=0$, and an explicit account of a discrete crystal lattice is necessary. However, account of the crystal lattice near the Bloch point core results in an energy correction, which is much smaller than the micromagnetic contribution if the lateral size of the Bloch point micromagnetic configuration is large compared to the lattice period^27^. In the article we apply the continuous micromagnetic field theory to describe Bloch point in a spherical ferromagnetic particle assuming that the small volume around the singularity (at temperature lower than the Curie point ${T}_{c}$) has essential influence neither on the Bloch point energy nor on the Hopf index. The same assumption was used in refs.^24,25,33^. The dependence ${M}_{s}\left(r\right)$ near the Curie point was accounted for in the papers^34–36^, when the longitudinal magnetic susceptibility is relatively large.

We performed micromagnetic simulations using the OOMMF code^48^. We used the magnetic parameters of a ferromagnetic sphere (the exchange stiffness $A=$ 21 pJ/m and saturation magnetization ${M}_{s}=$ 1700 kA/m) with different radii within the range 20–175 nm, and discretized the system with appropriate cell sizes to obtain a suitable resolution in our results:

**Table:** 

Sphere radius	Cell size
20 nm	0.2 × 0.2 × 0.2 nm^3^
30 nm	0.4 × 0.4 × 0.4 nm^3^
40 nm	0.5 × 0.5 × 0.5 nm^3^
Equal to or greater than 50 nm	1 × 1 × 1 nm^3^

In order to obtain the Bloch point state, we initially have placed two vortex core configurations with opposite polarities in the ± *z*-direction. This magnetic configuration is relaxed by minimizing the total energy $w$ (Eq. (7)) until a stable state is reached (see Fig. 2a). The conjugate gradient method was used in this process.

It is worth noting that different initial magnetization configurations were used for simulations, but not all of them reached a Bloch point state. Only when the size of the vortex core, used initially, is large enough (greater than 80% of the sphere radius, approximately), the Bloch point state is reached.
